# Subunit Vaccines Against Emerging Pathogenic Human Coronaviruses

**DOI:** 10.3389/fmicb.2020.00298

**Published:** 2020-02-28

**Authors:** Ning Wang, Jian Shang, Shibo Jiang, Lanying Du

**Affiliations:** ^1^Lindsley F. Kimball Research Institute, New York Blood Center, New York, NY, United States; ^2^Department of Veterinary and Biomedical Sciences, College of Veterinary Medicine, University of Minnesota, Saint Paul, MN, United States; ^3^Key Laboratory of Medical Molecular Virology (MOE/NHC/CAMS), School of Basic Medical Sciences, Shanghai Medical College, Fudan University, Shanghai, China

**Keywords:** human coronaviruses, pathogenesis, SARS-CoV, MERS-CoV, 2019-nCoV, subunit vaccines

## Abstract

Seven coronaviruses (CoVs) have been isolated from humans so far. Among them, three emerging pathogenic CoVs, including severe acute respiratory syndrome coronavirus (SARS-CoV), Middle East respiratory syndrome coronavirus (MERS-CoV), and a newly identified CoV (2019-nCoV), once caused or continue to cause severe infections in humans, posing significant threats to global public health. SARS-CoV infection in humans (with about 10% case fatality rate) was first reported from China in 2002, while MERS-CoV infection in humans (with about 34.4% case fatality rate) was first reported from Saudi Arabia in June 2012. 2019-nCoV was first reported from China in December 2019, and is currently infecting more than 70000 people (with about 2.7% case fatality rate). Both SARS-CoV and MERS-CoV are zoonotic viruses, using bats as their natural reservoirs, and then transmitting through intermediate hosts, leading to human infections. Nevertheless, the intermediate host for 2019-nCoV is still under investigation and the vaccines against this new CoV have not been available. Although a variety of vaccines have been developed against infections of SARS-CoV and MERS-CoV, none of them has been approved for use in humans. In this review, we have described the structure and function of key proteins of emerging human CoVs, overviewed the current vaccine types to be developed against SARS-CoV and MERS-CoV, and summarized recent advances in subunit vaccines against these two pathogenic human CoVs. These subunit vaccines are introduced on the basis of full-length spike (S) protein, receptor-binding domain (RBD), non-RBD S protein fragments, and non-S structural proteins, and the potential factors affecting these subunit vaccines are also illustrated. Overall, this review will be helpful for rapid design and development of vaccines against the new 2019-nCoV and any future CoVs with pandemic potential. This review was written for the topic of *Antivirals for Emerging Viruses: Vaccines and Therapeutics* in the *Virology* section of *Frontiers in Microbiology*.

## Introduction

**FIGURE 1:**
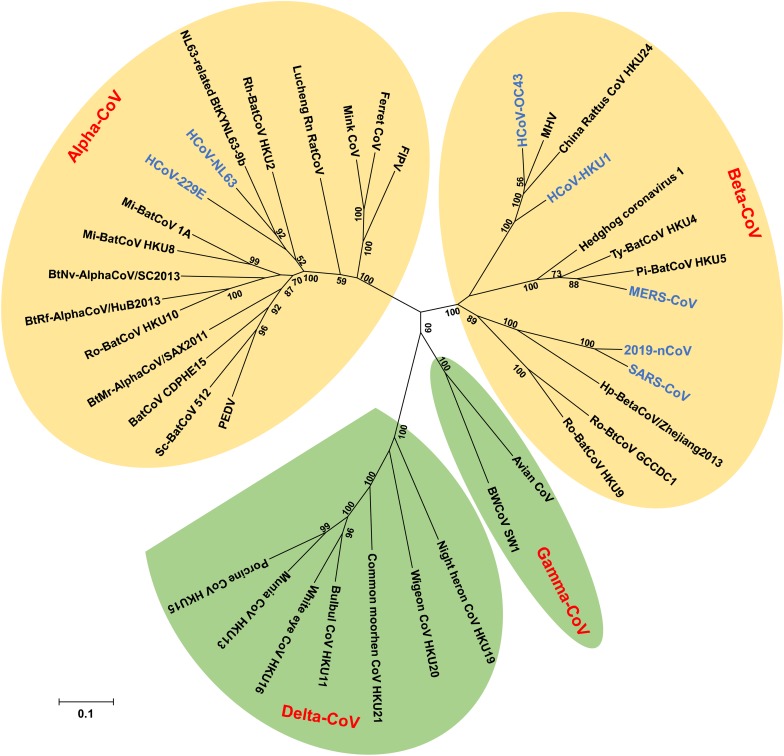
Phylogenetic tree of coronaviruses (CoVs) based on the nucleotide sequences of RNA dependent RNA polymerase (RdRp). The Tree, with 1,000 bootstrap values, was constructed by the maximum likelihood method using MEGA 6. The four main phylogenetic clusters correspond to genera alpha-CoV, beta-CoV, gamma-CoV, and delta-CoV. Each CoV genus contains different subgenera. The letters in blue indicate human CoVs.

Coronaviruses (CoVs) belong to the subfamily *Othocoronavirinae*, in the family *Coronaviridae* of the order *Nidovirales*. According to the 10^th^ Report on Virus Taxonomy from the International Committee on Taxonomy of Viruses (ICTV), the *Othocoronavirinae* is comprised of four genera, including *alphacoronavirus (alpha-CoV)*, *betacoronavirus (beta-CoV)*, *gammacoronavirus (gamma-CoV)*, and *deltacoronavirus (delta-CoV)* (King et al., 2018). Alpha- and beta-CoVs can infect mammals, including but not limited to bats, pigs, cats, mice, and humans (Kusanagi et al., 1992; Li et al., 2005b; Poon et al., 2005; Drexler et al., 2014; Pedersen, 2014; Kudelova et al., 2015; Cui et al., 2019). Gamma- and delta-CoVs usually infect birds, while some of them could infect mammals (Woo et al., 2009a, 2012, 2014; Ma et al., 2015). Since the late sixties, CoVs have been recognized as one of the viral sources responsible for the common cold. Among all CoVs identified so far, seven have the ability to infect humans, including human coronavirus 229E (HCoV-229E) and human coronavirus NL63 (HCoV-NL63), which belong to alpha-CoVs (Hamre and Procknow, 1966; Chiu et al., 2005), as well as human coronavirus OC43 (HCoV-OC43), human coronavirus HKU1 (HCoV-HKU1), severe acute respiratory syndrome coronavirus (SARS-CoV), Middle East respiratory syndrome coronavirus (MERS-CoV), and the newly emerged coronavirus (2019-nCoV), which are known to be beta-CoVs (Drosten et al., 2003; Ksiazek et al., 2003; Vabret et al., 2003; Woo et al., 2005; Zaki et al., 2012; Du et al., 2016b; Zhang et al., 2020; Zhu et al., 2020) (Figure 1).

Four human CoVs, including HCoV-229E, HCoV-NL63, HCoV-OC43, and HCoV-HKU1, have been identified in humans, but without causing severe infections. HCoV-229E was isolated from nasal secretions of medical students with minor upper respiratory disease. This virus was an original isolate, and was first reported in the 1960s (Hamre and Procknow, 1966). In addition to HCoV-229E, several studies have reported the recovery of HCoV-OC43 from patients with upper respiratory tract illness (Tyrrell and Bynoe, 1965; Hamre et al., 1967; McIntosh et al., 1967; Kapikian et al., 1969). In 2004, HCoV-NL63 was isolated from clinical species of infants suffering from pneumonia or bronchiolitis, and characterized for its ability to infect human respiratory tract (Fouchier et al., 2004; van der Hoek et al., 2004). The subsequent study in 2005 identified a new member of CoVs, named HCoV-HKU1, from a 71-year-old man with pneumonia (Woo et al., 2005). Generally, these four viruses are the most common pathogens causing mild upper respiratory infection or asymptomatic infection, and count for about 30% of all colds (Myint, 1994; Lau et al., 2006; Kim et al., 2017). In the serological surveillance on healthy adults, HCoV-229E, HCoV-NL63, and HCoV-OC43 demonstrated more than 90% seropositive with the immunological assay. It appears common for these CoVs to infect children (Mourez et al., 2007; Shao et al., 2007; Severance et al., 2008). In contrast to the above three human CoVs, HCoV-HKU1 has around 50% seropositive in healthy individuals and a relatively low exposure rate in children (Lehmann et al., 2008; Severance et al., 2008). Although the prevalence of various CoVs is different, the incidence among these viruses shows no significant difference (Woo et al., 2009b). The afore-mentioned four CoVs have been detected in 2.1–17.9% of clinical specimens (Esper et al., 2006; Lau et al., 2006; Gerna et al., 2007; Regamey et al., 2008; Matoba et al., 2015; Killerby et al., 2018). These viruses have also been associated with lower respiratory tract illness in children, elders, and immunodeficient individuals (Falsey et al., 2002; Fouchier et al., 2004; Woo et al., 2005; Gerna et al., 2006). HCoV-229E and HCoV-OC43 may lead to central nervous system infection since viral RNAs are detected in the brain of some patients (Arbour et al., 2000; Desforges et al., 2014).

Unlike the above four human CoVs, SARS-CoV, MERS-CoV, and 2019-nCoV have caused severe pneumonia and/or failure of other organs, even death, among infected populations (Nicholls et al., 2003; Zhong et al., 2003; Zaki et al., 2012; Zhu et al., 2020). The epidemic outbreak of SARS-CoV began in the Guangdong Province of China in November 2002, and spread through human-to-human transmission to other parts of the world within a few months (Ksiazek et al., 2003). From November 2002 to August 2003, SARS-CoV infected more than 8,098 people in 29 counties, resulting in over 774 deaths with ∼10% fatality rate (Du et al., 2009a). Palm civets serving as a potential intermediate host of this virus were traced immediately (Tu et al., 2004). Chinese horseshoe bats (*Rhinolophus sinicus*) are the natural reservoir of SARS-CoV (Li et al., 2005b). Various bat SARS-related CoVs (SARSr-CoV) have been identified in Yunnan, China, several of which can infect human cells, and have been further characterized (Ge et al., 2013; Hu et al., 2017). These discoveries indicate the threat of re-emergence of SARS-CoV or SARSr-CoV.

A decade later, another highly pathogenic human CoV, MERS-CoV, emerged, and the first patient with MERS-CoV infection was reported in Saudi Arabia in June 2012 (Zaki et al., 2012). By December 26, 2019, a total of 2,494 laboratory-confirmed cases of MERS, including 858 associated deaths in 27 countries (fatality rate 34.4%), were reported to the WHO^1^. Globally, the majority (about 80%) of human cases have been reported in Saudi Arabia, where people get infected through direct contact with infected dromedary camels or persons^2^ (Zaki et al., 2012). Isolation of MERS-CoV and detection of neutralizing antibodies from dromedary camels suggest that these camels are potentially an important intermediate host (Reusken et al., 2013; Azhar et al., 2014). Similar to SARS-CoV, MERS-CoV is also an emerging zoonotic virus (Li and Du, 2019). Bats habituate several CoVs phylogenetically related to MERS-CoV, and some of them are identical to MERS-CoVs, suggesting that MERS-CoV may originate from bats (Annan et al., 2013; Lelli et al., 2013; Lau et al., 2018; Luo et al., 2018a). Different from SARS-CoV, which has not caused infections in humans since 2004 (Du et al., 2009a), the transmission of MERS-CoV has not been interrupted, and the infected human cases continue increasing^1^ (Mobaraki and Ahmadzadeh, 2019). Currently, human-to-human transmission of MERS-CoV is limited.

A new CoV, 2019-nCoV, has caught worldwide attention (Liu and Saif, 2020; Zhang et al., 2020). It was first identified in Wuhan, China in December 2019, from patients with pneumonia (Zhu et al., 2020), and has infected more than 70000 people globally, including 2,009 deaths (∼2.7% fatality rate), as of February 19, 2020, particularly in China, and the other parts of the world, including Australia, Japan, Malaysia, Singapore, South Korea, Viet Nam, Cambodia, Philippines, Thailand, Nepal, Sri Lanka, India, United States, Canada, France, Finland, Germany, Italy, Russian Federation, Spain, Sweden, United Kingdom, Belgium, Egypt, and United Arab Emirates^3^. Different from MERS-CoV but similar to SARS-CoV, 2019-nCoV can cause human-to-human transmission, and its intermediate host that leads to the current human infection and outbreak is still under investigation.

## Genome of Emerging Human Coronaviruses, as Well as Structure and Function of Their Key Proteins

**FIGURE 2:**
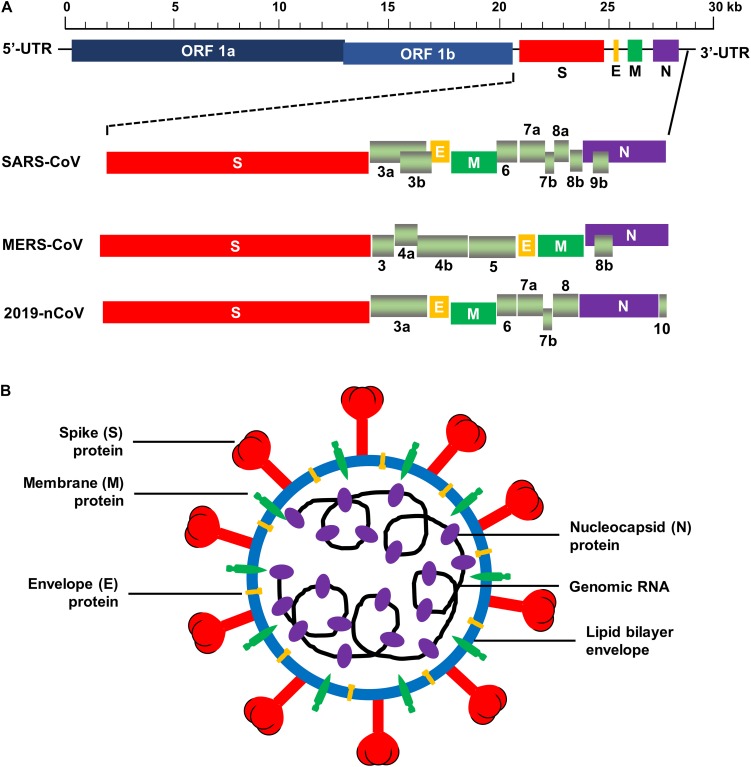
Schematic structure of SARS-CoV, MERS-CoV, and 2019-nCoV. **(A)** Schematic diagram of genomic organization of SARS-CoV, MERS-CoV, and 2019-nCoV. The genomic regions or open-reading frames (ORFs) are compared. Structural proteins, including spike (S), envelope (E), membrane (M) and nucleocapsid (N) proteins, as well as non-structural proteins translated from ORF 1a and ORF 1b and accessory proteins, including 3a, 3b, 6, 7a, 7b, 8a, 8b, and 9b (for SARS-CoV), 3, 4a, 4b, 5, and 8b (for MERS-CoV), and 3a, 6, 7a, 7b, 8, and 10 (for 2019-nCoV) are indicated. 5′-UTR and 3′-UTR, untranslated regions at the N- and C-terminal regions, respectively. Kb, kilobase pair. **(B)** Schematic structure of virion of SARS-CoV, MERS-CoV, and 2019-nCoV and its major structural proteins.

The human CoVs are enveloped viruses with a positive-sense, single-stranded RNA genome. They are 80–160 nm in diameter. Like other CoVs, human CoVs contain the largest viral genome [27–32 kilobase pairs (kb)] among the RNA viruses, and they share similar genome organization (Fehr and Perlman, 2015). Two large overlapping open reading frames (ORFs), *ORF 1a* and *ORF 1b*, occupy two-thirds of the genome at the 5′-terminus, and a third of the genome at the 3′-terminus encodes four common structural proteins in the gene order of spike (S), envelope (E), membrane (M), and nucleocapsid (N) (5′–3′) (Fehr and Perlman, 2015). The large *ORF 1ab* is a replicase gene encoding polyproteins 1a (pp1a) and pp1b/1ab, which can be cleaved into 15–16 non-structural proteins (nsp2-nsp16 or nsp1-nsp16) by 3C-like proteinase (3CL^pro^, nsp5) and papain-like proteinase (PL^pro^, nsp3) (Bailey-Elkin et al., 2014; Fehr and Perlman, 2015; Tomar et al., 2015; Snijder et al., 2016). In addition to the genes encoding the above structural proteins, the genes encoding accessory proteins have also been detected in the 3′ region between *S–E–M–N* (Fehr and Perlman, 2015). Some beta-CoVs, such as HCoV-OC43 and HCoV-HKU1, contain hemagglutinin-esterase (*HE*) gene located between *ORF 1ab* and *S* gene encoding an additional structural protein, HE (De Groot et al., 2011; Desforges et al., 2013; Huang et al., 2015). Similar to other human CoVs, SARS-CoV possesses a ∼29-kb genome, which encodes pp1a and pp1ab, four main structural proteins (S, E, M, and N), and eight accessory proteins, such as 3a, 3b, 6, 7a, 7b, 8a, 8b, and 9b (Figure 2A) (Marra et al., 2003; Snijder et al., 2003). The MERS-CoV genome is about 30 kb in length and encodes pp1a, pp1ab, four structural proteins (S, E, M, and N), and five accessory proteins (3, 4a, 4b, 5, and 8b) (Figure 2A) (van Boheemen et al., 2012). The genomic RNA of SARS-CoV and MERS-CoV is packed inside capsid formed by the N protein, while the M, E, and S proteins form the envelope surrounding the capsid (Figure 2B). Accessory genes may incorporate into virions at low levels (Liu et al., 2014). Nevertheless, neither SARS-CoV nor MERS-CoV appears to contain the *HE* gene (Rota et al., 2003; Zaki et al., 2012).

Several proteins of human CoVs are important for viral infection and/or pathogenesis. For example, most nsps participate in viral RNA replication and/or transcription (Snijder et al., 2016). The accessory proteins interact with host cells, potentially helping the viruses to evade the immune system and increase their virulence (Menachery et al., 2017). The HE protein assists in the attachment of virus–host cells, thus playing a key role in the production of infectious virions, as in the case of HCoV-OC43 (Desforges et al., 2013). The M and E proteins are responsible for virus assembly or promote virulence (Scobey et al., 2013; DeDiego et al., 2014). Different from the above proteins, the S protein of human CoVs mediates viral entry into host cells and subsequent membrane fusion, enabling viral infection (Du et al., 2009a; Lu et al., 2014). The S protein is a class I viral protein, which can be cleaved into two functional subunits, an amino-terminal S1 subunit and a carboxyl-terminal S2 subunit. The S1 subunit is responsible for virus–host cell receptor binding, whereas the S2 subunit is involved in virus–host membrane fusion (Li et al., 2005a; Lu et al., 2014). The S1 contains two major domains, an N-terminal domain (NTD) and a C-terminal domain (CTD). In general, NTDs mediate sugar binding (Li, 2016; Li et al., 2017; Ou et al., 2017; Hulswit et al., 2019; Tortorici et al., 2019), whereas CTDs facilitate protein receptor recognition (Wong et al., 2004; Hofmann et al., 2006; Lu et al., 2013). The NTDs and CTDs of the S1 subunit can bind host receptors or function as receptor-binding domains (RBDs) (Lu et al., 2013; Hulswit et al., 2019). The entry of human CoVs relies on the interaction between viral and cellular membrane proteins. Recognition of S1 subunit with a receptor and/or sugar on the cell surface initiates the infection (Li, 2015). After the initial recognition and binding, the S protein undergoes conformational changes, followed by membrane fusion through the S2 region (Li, 2015, 2016; Du et al., 2017). Consequently, the viral genetic materials are delivered into the host cell through the fusion core (Du et al., 2009a).

Similar to HCoV-NL63 (Hofmann et al., 2005; Li, 2015), SARS-CoV recognizes, through the RBD in the CTD region of its S1 subunit, angiotensin-converting enzyme 2 (ACE2) as the receptor on the target cell (Li et al., 2003). The RBD (CTD) in two states (standing or lying) has been observed in the trimeric S protein. ACE2 binds to standing RBD, specifically in the receptor-binding motif (RBM), keeping the RBD in the “standing” state (Yuan et al., 2017). In addition to human ACE2, SARS-CoV S protein could also bind to palm civet and mouse ACE2s (Li et al., 2004; Li, 2008). Mutations in the RBD of S1 subunit are required for cross-species transmission of SARS-CoV (Li et al., 2005c; Li, 2008, 2016). Several bat SARSr-CoVs have been identified, and these CoVs can utilize human ACE2 as their receptor to bind the target cells (Ge et al., 2013; Hu et al., 2017). The structure of SARS-CoV S trimer and RBD binding to the ACE2 receptor is shown in Supplementary Figures S1A,B.

MERS-CoV RBD shares a structure similar to that of the homology domain of SARS-CoV (Wang et al., 2013). However, antibodies induced by SARS-CoV RBD have no cross-reactivity and/or cross-neutralizing activity to MERS-CoV (Du et al., 2013b). Moreover, MERS-CoV utilizes dipeptidyl peptidase 4 (DPP4) as a receptor through the RBD (CTD) region (Raj et al., 2013), which is distinct from the SARS-CoV receptor ACE2. Although the core regions of SARS-CoV and MERS-CoV RBDs are similar, their RBM regions are significantly different, which explains why they recognize different receptors (Li, 2015). MERS-CoV S trimer also maintains a structure similar to that of SARS-CoV S trimer. Both standing and lying states can be detected in the MERS-CoV RBDs, whereas DPP4 only binds to the standing RBD (Pallesen et al., 2017; Yuan et al., 2017). MERS-CoV is clustered with Ty-BatCoV HKU4 and Pi-BatCoV HKU5 in the subgenus *Merbecovirus*. Ty-BatCoV HKU4, but not Pi-BatCoV HKU5, could use human DPP4 as a receptor (Yang et al., 2014b). Recently, some MERS-related CoVs (MERSr-CoVs) have been discovered from bats that can enter human DPP4-expressing cells (Lau et al., 2018; Luo et al., 2018a). These findings suggest that the emergence of MERSr-CoV may threaten human health owing to their potential for cross-species transmission. The MERS-CoV S protein and RBD and their complexes, along with the DPP4 receptor, are shown in Supplementary Figures S1C,D.

Recent studies have found that the new human CoV, 2019-nCoV, which belongs to the species of SARSr-CoV, shares high sequence identify (about 79.5%) to SARS-CoV (Zhou et al., 2020). The genome of 2019-nCoV encodes pp1ab (translated from ORF 1ab), four structural proteins (S, E, M, and N), and six accessory proteins (3a, 6, 7a, 7b, 8, and 10) (Figure 2A). Same as SARS-CoV and MERS-CoV, 2019-nCoV appears to have no *HE* gene. The virion of 2019-nCoV consists of similar structure as SARS-CoV and MERS-CoV (Figure 2B). Like SARS-CoV, 2019-nCoV also uses ACE2 as its cellular receptor to enter host cells (Zhou et al., 2020). Currently, the structure of 2019-nCoV RBD and/or its binding with the viral receptor has not yet been available.

## Overview of Vaccines Against Emerging Pathogenic Human Coronaviruses

Unlike the four low pathogenic human CoVs, including HCoV-229E, HCoV-NL63, HCoV-OC43, and HCoV-HKU1, which cause mild to no pathogenesis in humans, SARS-CoV, MERS-CoV, and 2019-CoV are three highly pathogenic human CoVs (Channappanavar and Perlman, 2017; Cui et al., 2019; Zhu et al., 2020). With the increasing numbers of 2019-nCoV and MERS-CoV infections and continuous threat of re-emergence of SARS-CoV, as well as the potential of SARS- and MERS-related CoVs to cause human infections, it is critical to develop vaccines with strong efficacy and safety targeting these viruses to prevent their infections in humans. Since the vaccines against 2019-nCoV have not been available, the rest of the review will focus on the vaccines against SARS-CoV and MERS-CoV.

Although a variety of vaccines have been developed against SARS-CoV and MERS-CoV, most of them are in the preclinical studies, and only several have been tested in clinical trials^4^ ^,5^ (Du et al., 2016b; Cho et al., 2018). Nevertheless, no vaccines have been approved for the prevention of SARS and MERS in humans, demonstrating the need to develop effective and safe vaccines to control current MERS-CoV infection, or to be stockpiled for potential use against re-emerged SARS-CoV or SARSr-CoV. Particularly, effective and safe vaccines are urgently needed to prevent and control the current outbreak of 2019-nCoV.

Most SARS-CoV and MERS-CoV vaccines developed thus far are based on the inactivated or live attenuated viruses, DNAs, proteins, nanoparticles, viral vectors, including virus-like particles (VLPs) (Zeng et al., 2004; Jiang et al., 2005; Liu et al., 2005; Du et al., 2009a, 2016b; Pimentel et al., 2009; Al-Amri et al., 2017). Each vaccine type has different advantages and disadvantages. For instance, inactivated and live-attenuated virus-based vaccines are vaccine types developed using the most traditional approaches. Although they generally induce highly potent immune responses and/or protection, the possibility for incomplete inactivation of viruses or recovering virulence exists, resulting in significant safety concerns (Zhang et al., 2014). Also, these traditional vaccines may induce the antibody-dependent enhancement (ADE) effect, as in the case of SARS-CoV infection (Luo et al., 2018b). Similarly, some viral-vectored vaccines can elicit specific antibody and cellular immune responses with neutralizing activity and protection, but they might also induce anti-vector immunity or present pre-existing immunity, causing some harmful immune responses. Instead, DNA and nanoparticle vaccines maintain strong safety profile; however, the immunogenicity of these vaccines is usually lower than that of virus- or viral vector-based vaccines, often requiring optimization of sequences, components, or immunization routes, inclusion of appropriate adjuvants, or application of combinational immunization approaches (Zhang et al., 2014).

## Subunit Vaccines Against Sars-CoV and Mers-CoV

Subunit vaccines are vaccines developed based on the synthetic peptides or recombinant proteins. Unlike inactivated or live-attenuated virus and some viral vectored vaccines, this vaccine type mainly contains specific viral antigenic fragments, but without including any components of infectious viruses, eliminating the concerns of incomplete inactivation, virulence recovery, or pre-existing immunity (Du et al., 2008; Deng et al., 2012). Similar to DNA or VLP-based vaccines, subunit vaccines are generally safe without causing potential harmful immune responses, making them promising vaccine candidates. Moreover, subunit vaccines may target specific, well-defined neutralizing epitopes with improved immunogenicity and/or efficacy (Du et al., 2008; Zhang et al., 2014).

A number of subunit vaccines against SARS-CoV and MERS-CoV have been developed, and these are described in detail in the next paragraphs. The targets used for the development of SARS-CoV and MERS-CoV subunit vaccines are also be discussed.

### Potential Targets for Development of SARS-CoV and MERS-CoV Subunit Vaccines

The S protein of SARS-CoV and MERS-CoV plays a vital role in receptor binding and membrane fusion. Thus, the S protein, but not other structural proteins, is the major antigen to induce protective neutralizing antibodies to block viruses from binding their respective receptor and thus inhibit viral infection (Bisht et al., 2004; Buchholz et al., 2004; Bukreyev et al., 2004; Yang et al., 2004). As a result, the S protein is also a major target for the development of subunit vaccines against SARS-CoV and MERS-CoV. Both full-length S protein and its antigenic fragments, including S1 subunit, NTD, RBD, and S2 subunit, can serve as important targets for the development of subunit vaccines (Guo et al., 2005; Mou et al., 2013; Wang et al., 2015; Jiaming et al., 2017; Zhou et al., 2018).

Although subunit vaccines based on the full-length S protein may elicit potent immune responses and/or protection, studies have found that antibodies induced by some of these vaccines mediate enhancement of viral infection *in vitro*, as in the case of SARS-CoV (Kam et al., 2007; Jaume et al., 2012), raising safety concerns for the development of full-length S protein-based subunit vaccines against SARS-CoV and MERS-CoV. In contrast, RBD-based subunit vaccines comprise the major critical neutralizing domain (Du and Jiang, 2015; Zhou et al., 2019). Therefore, these vaccines may generate potent neutralizing antibodies with strong protective immunity against viral infection. S1 subunit, for example, is much shorter than the full-length S protein, but it is no less able to induce strong immune responses and/or protection against viral infection (Li et al., 2013; Adney et al., 2019). Thus, this fragment can be used as an alternative target for subunit vaccine development. Despite their ability to induce immune responses and/or neutralizing antibodies, NTD and S2 as the targets of subunit vaccines are less immunogenic, eliciting significantly lower antibody titers, cellular immune responses, and/or protection than the other regions, such as full-length, S1, and RBD (Guo et al., 2005; Jiaming et al., 2017). Therefore, in terms of safety and efficacy, the RBD and/or S1 of S protein could be applied as critical targets for the development of subunit vaccine candidates against SARS-CoV, MERS-CoV, SARSr-CoV, and MERSr-CoV. Because of its conserved amino acid sequences and high homology among different virus strains (Elshabrawy et al., 2012; Zhou et al., 2018), the S2 subunit has potential to be used as a target for the development of universal vaccines against divergent virus strains.

In addition to the S protein, the N protein of SARS-CoV and MERS-CoV may serve as an additional target for the development of subunit vaccines. Unlike S protein, the N protein has no ability to elicit neutralizing antibodies to block virus-receptor interaction and neutralize viral infection, but it may induce specific antibody and cellular immune responses (Liu et al., 2006; Zheng et al., 2009). Several immunodominant B-cell and T-cell epitopes have been identified in the N protein of SARS-CoV and MERS-CoV, some of which are conserved in mice, non-human primates, and humans (Liu et al., 2006; Chan et al., 2011; Veit et al., 2018). Other proteins, such as M protein, can be used as potential targets of SARS-CoV and MERS-CoV subunit vaccines. Notably, SARS-CoV M protein-derived peptides have immunogenicity to induce high-titer antibody responses in the immunized animals (He et al., 2005b), suggesting the potential for utilizing this protein to develop subunit vaccines.

### Subunit Vaccines Against SARS-CoV

Numerous subunit vaccines against SARS-CoV have been developed since the outbreak of SARS, the majority of which use the S protein and/or its antigenic fragments, in particular, RBD, as the vaccine target (Table 1).

**TABLE 1:** Subunit Vaccines against SARS-CoV^a^.

Name	Antigenicity and functionality	Adjuvant	Route	Animal models	Antibody response	Cellular immune response	Protection	References
**Subunit vaccines based on SARS-CoV full-length or trimeric S protein**								
FL-S and EC-S proteins	Bind to SARS-CoV S1, NTD, RBD, and S2-specific mAbs	MPL + TDM	S.C.	BALB/c mice	Elicit SARS-CoV S-specific Abs (IgG, > 1: 2 × 10^5^), neutralizing (> 1:2.4 × 10^4^) pseudotyped SARS-CoV (Tor2, GD03, and SZ3 strains)	N/A	N/A	He et al., 2006a
S andS-foldon proteins	N/A	TiterMax Gold; Alum Hydro+MPL	S.C. or I.M.	BALB/c mice	Elicit SARS-CoV S-specific Abs (IgG, > 1:10^4^) in mice, neutralizing (∼2.4 × 10^2^ for S; ∼1:7 × 10^2^ for S-foldon) live SARS-CoV (Urbani strain)	N/A	Protect vaccinated mice from challenge of SARS-CoV (Urbani strain, 10^5^ TCID_50_) with undetectable viral load in lungs	Li et al., 2013
triSpike protein	N/A	Alum hydro	I.P. or S.C.	BALB/c mice; Hamsters	Elicits SARS-CoV S-specific mucosal and serum Abs (IgA and IgG) in mice and hamsters, blocking S-ACE2 receptor binding and neutralizing live SARS-CoV (HKU-39849 strain); induces ADE	N/A	Protects vaccinated hamsters from challenge of SARS-CoV (Urbani strain, 10^3^ TCID_50_) with undetectable or reduced viral load in lungs	Kam et al., 2007; Jaume et al., 2012
**Subunit vaccines based on SARS-CoV RBD protein**								
RBD-Fc protein	N/A	Freund’s	I.D. or I.M.	BALB/c mice; Rabbits	Elicits SARS-CoV S/RBD-specific Abs (IgG) in mice and rabbits, neutralizing pseudotyped (rabbits: ≥ 7.3 × 10^4^) and live (mice: 1:4 × 10^3^; rabbits: > 1:1.5 × 10^4^) SARS-CoV (BJ01 strain)	N/A	Protects majority (4/5) of vaccinated mice from challenge of SARS-CoV (BJ01 strain, 10^6^ TCID_50_), with one mouse showing mild alveolar damage in lungs	He et al., 2004; Du et al., 2007
RBD193-CHO; RBD219-CHO proteins	Binds to SARS-CoV RBD-specific mAbs (neutralizing 24H8, 31H12, 35B5, 33G4, 19B2; non-neutralizing 17H9)	Freund’s	S.C.	BALB/c mice	Elicit SARS-CoV RBD-specific Abs, neutralizing pseudotyped (< 1:10^4^ for RBD193-CHO; 1:5.8 × 10^4^ for RBD219-CHO) and live (< 1:10^3^ for RBD193-CHO; 1:10^3^ for RBD219-CHO) SARS-CoV (GZ50 strain)	Induce SARS-CoV RBD-specific cellular immune responses (IFN-γ, IL-2, IL-4, IL-10) in mice	Protect all (for RBD219-CHO) or majority (3/5, for RBD219-CHO) of vaccinated mice from challenge of SARS-CoV (GZ50 strain, 100 TCID_50_ for RBD193-CHO; 5 × 10^5^ TCID_50_ for RBD219-CHO) with undetectable viral RNA or no, to reduced, viral load in lungs	Du et al., 2009c, 2010
RBD-293T protein	Binds to SARS-CoV RBD-specific mAbs (neutralizing 24H8, 31H12, 35B5, 33G4, 19B2; non-neutralizing 17H9)	SAS	S.C.	BALB/c mice	Elicits SARS-CoV RBD-specific Abs (IgG), neutralizing pseudotyped (1:6.9 × 10^5^) and live (1:1.6 × 10^3^) SARS-CoV (GZ50 strain)	N/A	Protects all vaccinated mice from challenge of SARS-CoV (GZ50 strain, 100 TCID_50_) with undetectable viral RNA and viral load in lungs	Du et al., 2009b
S318-510 protein	N/A	Alum; Alum + CpG	S.C.	129S6/SvEv mice	Elicits SARS-CoV-specific Abs (IgG, IgG1, and IgG2a) in mice. Reduces neutralization after removing glycosylation	Induces SARS-CoV S-specific cellular immune responses (IFN-γ) in mice	N/A	Zakhartchouk et al., 2007
**Subunit vaccines based on non-RBD SARS-CoV S protein fragments**								
S1 and S1-foldon proteins	N/A	TiterMax Gold; Alum Hydro + MPL	S.C. or I.M.	BALB/c mice	Elicit SARS-CoV S-specific Abs (IgG, > 1:10^4^) in mice, neutralizing (1:1.7 × 10^2^ for S1; 1:90 for S1-foldon) live SARS-CoV (Urbani strain)	N/A	Protect vaccinated mice from challenge of SARS-CoV (Urbani strain, 10^5^ TCID_50_) with undetectable viral load in lungs	Li et al., 2013
S2 protein	N/A	Freund’s	S.C.	BALB/c mice	Elicits SARS-CoV S2-specific Abs (IgG, 1:1.6 × 10^3^) in mice with no neutralizing activity	Induces SARS-CoV S2-specific cellular immune responses (IFN-γ and IL-4) in mice	N/A	Guo et al., 2005
**Subunit vaccines based on SARS-CoV non-S structural proteins (i.e. N and M)**								
rN protein	N/A	Freund’s	I.P.	BALB/c mice	Elicits SARS-CoV N-specific Abs (IgG (1:1.8 × 10^3^), IgG1, and IgG2a) in mice	Induces cellular immune responses with up-regulated IFN-γ and IL-10 cytokines in mice	N/A	Zheng et al., 2009
rN protein	N/A	Montanide + CpG; Freund’s	S.C.	BALB/c mice	Elicits SARS-CoV N-specific Abs (IgG) in mice	Induces SARS-CoV N-specific cellular immune responses (IFN- γ) in mice	N/A	Liu et al., 2006
M1-31 and M132-161 peptides	Bind to sera from SARS patients or immunized mice and rabbits	Freund’s	I.D.	BALB/c mice; NZW rabbits	Induce SARS-CoV M-specific Abs (IgG) in rabbits	N/A	N/A	He et al., 2005b

*^*a*^Abs, antibodies; ADE, antibody-dependent enhancement; Alum hydro, aluminum hydroxide; CHO, Chinese hamster ovary; CpG, cysteine-phosphate-guanine; I.D., intradermal; I.M., intramuscular; IFN-γ, interferon gamma; IL-2, interleukin 2; IL-4, interleukin 4; IL-10, Interleukin 10; I.P., intraperitoneal; mAbs, monoclonal antibodies; Montanide, Montanide ISA-51; MPL + TDM, monophosphoryl lipid A and trehalose dicorynomycolate; N/A, not reported; NTD, N-terminal domain; NZW rabbits, New Zealand White rabbits; RBD, receptor-binding domain; SAS, Sigma adjuvant system; S.C., subcutaneous; TCID_50_, median tissue culture infectious dose.*

#### SARS-CoV Subunit Vaccines Based on Full-Length S Protein

Subunit vaccines based on SARS-CoV S protein, including full-length or trimeric S protein, are immunogenic with protection against SARS-CoV infection (He et al., 2006a; Kam et al., 2007; Li et al., 2013). Either insect cell-expressed full-length (FL-S) or extracellular domain (EC-S) SARS-CoV S protein developed high-titer S-specific antibodies with neutralizing activity against pseudotyped SARS-CoV expressing S protein of representative SARS-CoV human and palm civet strains (Tor2, GD03, and SZ3) isolated during the 2002 and 2003 or 2003 and 2004 outbreaks (He et al., 2006a). In addition, full-length S-ectodomain proteins fused with or without a foldon trimeric motif (S or S-foldon) could elicit specific antibody responses and neutralizing antibodies, protecting immunized mice against SARS-CoV challenge with undetectable virus titers in the lungs (Li et al., 2013). Moreover, a subunit vaccine (triSpike) based on a full-length S protein trimer induced specific serum and mucosal antibody responses and efficient neutralizing antibodies against SARS-CoV infection (Kam et al., 2007). Nevertheless, this vaccine also resulted in Fcγ receptor II (FcγRII)-dependent and ACE2-independent ADE, particularly in human monocytic or lymphoblastic cell lines infected with pseudotyped SARS-CoV expressing viral S protein, or in Raji B cells (B-cell lymphoma line) infected with live SARS-CoV (Kam et al., 2007; Jaume et al., 2012), raising significant concerns over the use of full-length S protein as a SARS vaccine target.

#### SARS-CoV Subunit Vaccines Based on RBD

SARS-CoV RBD contains multiple conformation-dependent epitopes capable of eliciting high-titer neutralizing antibodies; thus, it is a major target for the development of SARS vaccines (He et al., 2004, 2005a; Jiang et al., 2012; Zhu et al., 2013). Subunit vaccines based on the SARS-CoV RBD have been extensively explored. Studies have found that a fusion protein containing RBD and the fragment crystallizable (Fc) region of human IgG1 (RBD-Fc) elicited highly potent neutralizing antibodies against SARS-CoV in the immunized rabbits and mice, which strongly blocked the binding between S1 protein and SARS-CoV receptor ACE2 (He et al., 2004). This RBD protein induced long-term, high-level SARS-CoV S-specific antibodies and neutralizing antibodies that could be maintained for 12 months after immunization, protecting most of the vaccinated mice against SARS-CoV infection (Du et al., 2007). In addition, recombinant RBDs (residues 318–510 or 318–536) stably or transiently expressed in Chinese hamster ovary (CHO) cells bound strongly to RBD-specific monoclonal antibodies (mAbs), elicited high-titer anti-SARS-CoV neutralizing antibodies, and protected most, or all, of the SARS-CoV-challenged mice, with undetectable viral RNA and undetectable or significantly reduced viral load (Du et al., 2009c, 2010). Significantly, a 293T cell-expressed RBD protein maintains excellent conformation and good antigenicity to bind SARS-CoV RBD-specific neutralizing mAbs. It elicited highly potent neutralizing antibodies that completely protected immunized mice against SARS-CoV challenge (Du et al., 2009b). Particularly, RBDs from the S proteins of Tor2, GD03, and SZ3, representative strains of SARS-CoV isolated from human 2002–2003, 2003–2004, and palm civet strains, can induce high-titer cross-neutralizing antibodies against pseudotyped SARS-CoV expressing respective S proteins (He et al., 2006c). Different from the full-length S protein-based SARS subunit vaccines, no obvious pathogenic effects have been identified in the RBD-based SARS subunit vaccines (Kam et al., 2007; Jaume et al., 2012).

#### SARS-CoV Subunit Vaccines Based on Non-RBD S Protein Fragments

SARS subunit vaccines based on S protein fragments (S1 and S2), other than the RBD, have shown immunogenicity and/or protective efficacy against SARS-CoV infection (Guo et al., 2005; Li et al., 2013). For example, recombinant S1 proteins fused with or without foldon elicited specific antibodies with neutralizing activity that protected immunized mice against high-dose SARS-CoV challenge (Li et al., 2013). Although some studies have demonstrated that recombinant SARS-CoV S2 (residues 681–980) protein elicits specific non-neutralizing antibody response in mice (Guo et al., 2005), others have indicated that mAbs targeting highly conserved heptad repeat 1 (HR1) and HR2 domains of SARS-CoV S protein have broad neutralizing activity against pseudotyped SARS-CoV expressing S protein of divergent strains (Elshabrawy et al., 2012), indicating the potential of utilizing the S2 region as a broad-spectrum anti-SARS-CoV vaccine target (Zheng et al., 2009).

#### SARS-CoV Subunit Vaccines Based on Non-S Structural Proteins

Subunit vaccines based on the N and M proteins of SARS-CoV have shown immunogenicity in vaccinated animals (Liu et al., 2006; Zheng et al., 2009). Studies have revealed that a plant-expressed SARS-CoV N protein conjugated with Freund’s adjuvant elicited specific IgG antibodies, including IgG1 and IgG2a subtypes, and cellular immune responses in mice, whereas another *E. coli*-expressed N protein conjugated with Montanide ISA-51 and cysteine-phosphate-guanine (CpG) adjuvants induced specific IgG antibodies toward a Th1 (IgG2a)-type response in mice (Liu et al., 2006; Zheng et al., 2009). Although N-specific antibodies have been detected in convalescent-phase SARS patient and immunized rabbit sera, they have no neutralizing activity against SARS-CoV infection (Qiu et al., 2005). In addition, immunodominant M protein peptides (M1-31 and M132-161) identified using convalescent-phase sera of SARS patients and immunized mouse and rabbit sera have immunogenicity to elicit specific IgG antibodies in rabbits (He et al., 2005b). In spite of their immunogenicity, it appears that these N- and M-based SARS subunit vaccines have not been investigated for their protective efficacy against SARS-CoV infection. Thus, it is unclear whether these non-S structural protein-based SARS subunit vaccines can prevent SARS-CoV infection.

#### Potential Factors Affecting SARS-CoV Subunit Vaccines

A number of factors may affect the expression of proteins to be used as SARS subunit vaccines; apart from their immunogenicity and/or protective efficacy. Understanding of these factors is important to generate subunit vaccines with good quality, high immunogenicity, and excellent protection against SARS-CoV infection.

The expression of recombinant protein-based SARS subunit vaccines may be changed by the following factors. First, addition of an intron splicing enhancer to the truncated SARS-CoV S protein fragments results in better enhancement of protein expression in mammalian cells than the exon splicing enhancers, and different cells may result in different fold increase of protein expression (Chang et al., 2006). Second, inclusion of a post-transcriptional gene silencing suppressor p19 protein from tomato bushy stunt virus to a SARS-CoV N protein may significantly increase its transient expression in tobacco (Zheng et al., 2009).

The following factors may affect the immunogenicity and protective efficacy of protein-based SARS subunit vaccines, including same proteins expressed in different expression systems, and same proteins with various lengths, amino acid mutations, or deletions (He et al., 2006b; Du et al., 2009b). For example, RBD proteins containing different lengths (193-mer: RBD193-CHO or 219-mer: RBD219-CHO) elicited different immune responses and protective efficacy against SARS-CoV challenge (Du et al., 2009c, 2010). A recombinant SARS-CoV RBD (RBD-293T) protein expressed in mammalian cell system was able to induce stronger neutralizing antibody response than those expressed in insect cells (RBD-Sf9) and *E. coli* (RBD-Ec) (Du et al., 2009b), suggesting that RBD purified from mammalian cells has preference for further development due to its ability to maintain native conformation. Notably, a single mutation (R441A) in the RBD of SARS-CoV disrupted its major neutralizing epitopes and affinity to bind viral receptor ACE2, thus abolishing the vaccine’s immunogenicity, and hence, its ability to induce neutralizing antibodies in immunized animals (He et al., 2006b). Additionally, deletion of a particular amino acid by changing a glycosylation site in the SARS-CoV RBD (RBD219-N1) also resulted in the alteration of subunit vaccine’s immunogenicity (Chen et al., 2014).

Other factors that potentially affect the immunogenicity of SARS subunit vaccines include immunization routes and adjuvants (Zakhartchouk et al., 2007; Li et al., 2013). Significantly high-titer antibodies were induced by monomeric or trimeric SARS-CoV S and S1 proteins through the intramuscular (I.M.) route compared to the subcutaneous (S.C.) route (Li et al., 2013). Moreover, a SARS-CoV RBD subunit vaccine conjugated with Alum plus CpG adjuvants elicited a higher level of IgG2a antibody and interferon gamma (IFN-γ) secretion than the RBD with Alum alone (Zakhartchouk et al., 2007).

### Subunit Vaccines Against MERS-CoV

Subunit vaccines against MERS-CoV have been developed extensively, almost all of which are based on the S protein, including full-length S timer, NTD, S1, and S2, particularly RBD. These subunit vaccines, including their antigenicity, functionality, immunogenicity, and protective efficacy in different animal models, are summarized in Table 2.

**TABLE 2:** Subunit Vaccines against MERS-CoV^a^.

Name	Functionality and antigenicity	Adjuvant	Route	Animal models	Antibody response	Cellular immune response	Protective efficacy	References
**Subunit vaccines based on MERS-CoV full-length S protein**								
MERS S-2P protein	Binds to DPP4 receptor and MERS-CoV S-NTD, RBD, and S2-specific neutralizing mAbs (G2, D12, and G4, respectively)	SAS	I.M.	BALB/c mice	Elicits neutralizing Abs in mice, neutralizing 7 pseudotyped MERS-CoV	N/A	N/A	Pallesen et al., 2017
**Subunit vaccines based on MERS-CoV RBD protein**								
rRBD (S367-606) protein	N/A	Alum Hydro + CpG or poly(I:C); IFA + CpG (mouse); Alum (NHPs)	I.M. or S.C.	BALB/c mice; NHPs	Elicits MERS-CoV RBD-specific Abs in mice (IgG, IgG1, IgG2a, and IgG2b) and NHPs (IgG), neutralizing pseudotyped (mouse: < 1:5 × 10^2^) and live (NHPs: < 1:5 × 10^2^) MERS-CoV (EMC2012 strain)	Induces MERS-CoV RBD-specific cellular immune responses (IFN-γ, TNF-α, IL-2, IL-4, IL-6, and IL-10) in mice and/or monkeys	Partially protects vaccinated NHPs from challenge of MERS-CoV (EMC2012 strain, 6.5 × 10^7^ TCID_50_) with alleviated pneumonia and decreased viral load	Lan et al., 2014, 2015
RBD (S377-662)-Fc protein	Binds to DPP4 receptor	Poly(I:C); Montanide	I.N. or S.C.	BALB/c mice	Elicits MERS-CoV S1- and RBD-specific Abs (IgA, IgG (> 1:10^4^), IgG1, IgG2a, and IgG3) in mice, neutralizing (≥ 1:2.4 × 10^2^) live MERS-CoV (EMC2012 strain)	Induces MERS-CoV S1-specific cellular immune responses (IFN-γ and IL-2) in mice	N/A	Du et al., 2013c; Ma et al., 2014b
RBD (S377-588)-Fc protein	Binds to DPP4 receptor and MERS-CoV RBD specific neutralizing mAbs (Mersmab1, m336, m337, and m338)	Montanide; MF59; AddaVax	I.M. or S.C.	BALB/c mice; hDPP4-Tg mice; Rabbits	Elicits MERS-CoV S1 and RBD-specific Abs in mice (IgG (> 1:10^5^), IgG1, and IgG2a) and rabbits (IgG), neutralizing 17 pseudotyped (≥ 1:10^4^) and 2 live (≥ 1:10^3^) MERS-CoV (EMC2012 and London1-2012 strains)	Induces MERS-CoV S1-specific cellular immune responses (IFN-γ and IL-2) in mice	Protects vaccinated Ad5/hDPP4-transduced BALB/c mice and majority (4/6) of vaccinated hDPP4-Tg mice from MERS-CoV (EMC2012 strain, 10^5^ PFU for BALB/c; 10^3–4^ TCID_50_ for Tg) challenge, without immunological toxicity or eosinophilic immune enhancement	Du et al., 2013a; Ma et al., 2014b; Tang et al., 2015; Zhang et al., 2016; Nyon et al., 2018
RBD-Fd protein	Binds to DPP4 receptor and MERS-CoV RBD-specific neutralizing mAbs (Mersmab1, m336, m337, and m338)	MF59; Alum	I.M. or S.C.	BALB/c mice; hDPP4-Tg mice	Elicits MERS-CoV S1-specific Abs (IgG (> 1:10^5^), IgG1, and IgG2a) in mice, neutralizing at least 9 pseudotyped (> 1:10^4^) and live (> 1:10^3^) MERS-CoV (EMC2012 strain)	N/A	Protects majority (5/6) of vaccinated hDPP4-Tg mice from challenge of MERS-CoV (EMC2012 strain, 10^4^ TCID_50_)	Tai et al., 2016
RBD (T579N) protein	Binds to receptor DPP4 and MERS-CoV RBD-specific neutralizing mAbs (hMS-1, m336, m337, and m338)	Montanide; Alum	I.M. or S.C.	BALB/c mice; hDPP4-Tg mice	Elicits neutralizing Abs (> 1:3 × 10^3^) in mice against live MERS-CoV (EMC2012 strain)	N/A	Protects all vaccinated hDPP4-Tg mice from challenge of MERS-CoV (EMC2012 strain, 10^4^ TCID_50_)	Du et al., 2016a
**Subunit vaccines based on non-RBD MERS-CoV S protein fragments**								
S1 protein	N/A	Ribi; Alum pho	I.M.	BALB/c mice; NHPs	Elicits MERS-CoV S1-specific Abs in mice (IgG and IgG1) and NHPs (IgG), neutralizing 8 pseudotyped and live MERS-CoV (JordanN3 strain)	N/A	Protects vaccinated NHPs from challenge of MERS-CoV (JordanN3 strain, 5 × 10^6^ PFU) with reduced abnormalities on chest CT	Wang et al., 2015
S1 protein	N/A	Advax + SAS	I.M.	Dromedary camels; Alpacas	Elicits neutralizing Abs in dromedary camels (≥ 1:80) and alpacas (≥ 1:6.4 × 10^2^) against live MERS-CoV (EMC2012 strain)	N/A	Protects vaccinated dromedary camels and alpacas from challenge of MERS-CoV (EMC2012 strain, 10^7^ TCID_50_) with reduced and delayed viral shedding in the upper airways (in camels) or complete protection (in alpacas)	Adney et al., 2019
rNTD protein	N/A	Alum pho + CpG	I.M.	BALB/c mice; Ad5-hDPP4 mice	Elicits MERS-CoV S-NTD-specific Abs (IgG, ≥ 1:10^4^) in mice, neutralizing pseudotyped and live (1:40) MERS-CoV (EMC2012 strain)	Induces MERS-CoV S-NTD-specific cellular immune responses (IFN-γ, IL-2, IL-6, IL-10, and IL-17A) in mice	Protects vaccinated Ad5-hDPP4-transduced mice from challenge of MERS-CoV (EMC2012 strain, 10^5^ PFU) with reduced lung abnormalities and respiratory tract pathology	Jiaming et al., 2017
SP3 peptide (aa736-761)	N/A	Freund’s	N/A	BALB/c mice; NZW rabbits	Elicits MERS-CoV S-specific Abs (IgG, 1:10^4^) in rabbits, neutralizing pseudotyped MERS-CoV	N/A	N/A	Yang et al., 2014a

*^*a*^aa, amino acid; Abs, antibodies; Ad5, adenovirus serotype 5; Ad5-hDPP4 mice, Ad5-hDPP4-transuced mice; Alum hydro, aluminum hydroxide; Alum pho, Aluminum phosphate; hDPP4, human dipeptidyl peptidase 4; hDPP4-Tg mice, transgenic mice expressing MERS-CoV receptor human DPP4; IFA, incomplete Freund’s adjuvant; I.M., intramuscular; I.N., intranasal; mAbs, monoclonal antibodies; Montanide, Montanide ISA51; N/A, not reported; NHPs, non-human primates; NZW, rabbits, New Zealand White rabbits; PFU, plaque-forming unit; rRBD, recombinant RBD; SAS, Sigma Adjuvant System; S.C., subcutaneous; TCID_50_, median tissue culture infectious dose; TNF-α, tumor necrosis factor (TNF)-alpha.*

#### MERS-CoV Subunit Vaccines Based on Full-Length S Protein

Subunit vaccines based on the full-length S protein cover both RBD and non-RBD neutralizing epitopes, some of which may be located in the conserved S2 subunit; thus this type of subunit vaccines are expected to induce high-titer neutralizing antibodies. Although several MERS-CoV full-length S protein-based vaccines have been reported in other vaccine types, including viral vectors and DNAs (Wang et al., 2015; Wang C. et al., 2017; Haagmans et al., 2016; Zhou et al., 2018), only a few subunit vaccines have been developed that rely on the full-length S protein. For example, a recombinant MERS-CoV S protein trimer (MERS S-2P) in prefusion conformation binds to the DPP4 receptor, as well as to the MERS-CoV NTD, RBD, and S2-specific neutralizing mAbs (Pallesen et al., 2017). Whereas this protein induces neutralizing antibodies in mice against divergent pseudotyped MERS-CoV *in vitro*, its *in vivo* protective activity against MERS-CoV infection is unknown (Pallesen et al., 2017). Therefore, more studies are needed to elucidate the potential for the development of MERS-CoV full-length S-based subunit vaccines, including understanding their protective efficacy and identifying possible harmful immune responses.

#### MERS-CoV Subunit Vaccines Based on RBD

Numerous MERS-CoV RBD-based subunit vaccines have been developed and extensively evaluated in available animal models since the emergence of MERS-CoV (Table 2) (Du et al., 2013c; Tai et al., 2017; Zhou et al., 2018). In general, these subunit vaccines have strong immunogenicity and are capable of inducing high neutralizing antibodies and/or protection against MERS-CoV infection (Ma et al., 2014b; Zhang et al., 2016; Tai et al., 2017; Wang Y. et al., 2017). Most subunit vaccines based on the MERS-CoV RBD have been described in detail in a previous review article (Zhou et al., 2019). In this section, we will briefly introduce these RBD-targeting MERS vaccines, and compare their functionality, antigenicity, immunogenicity, and protection against MERS-CoV infection.

Co-crystallographic analyses of MERS-CoV RBD and/or RBD/DPP4 complexes have confirmed that the RBD is attributed to residues 367–588 (Chen et al., 2013) or 367–606 (Lu et al., 2013) in the MERS-CoV S1 subunit. Indeed, a recombinant MERS-CoV RBD (rRBD) fragment (residues 367–606) elicits RBD-specific antibody and cellular immune responses and neutralizing antibodies in mice and/or non-human primates (NHPs) (Lan et al., 2014, 2015). However, it only partially protects NHPs from MERS-CoV infection by alleviating pneumonia and clinical manifestations, as well as decreasing viral load (Lan et al., 2015). In addition, an RBD protein fragment containing MERS-CoV S residues 377–622 fused with the Fc tag of human IgG can induce MERS-CoV S1- and/or RBD-specific humoral and cellular immune responses in the immunized mice with neutralizing activity against MERS-CoV infection (Du et al., 2013c; Jiang et al., 2013). However, after comparing several versions of MERS-CoV RBD fragments with different lengths, it was found that a truncated RBD (residues 377–588) had the highest DPP4-binding affinity and induced the highest-titer IgG antibodies and neutralizing antibodies against MERS-CoV, identifying its role as a critical neutralizing domain (Ma et al., 2014b).

Subsequently, several MERS-CoV subunit vaccines have been designed based on the identified critical neutralizing domain of RBD fragment, including those expressed in a stable CHO cell line (S377-588-Fc), fusing with a trimeric motif foldon (RBD-Fd), or containing single or multiple mutations in the RBD of representative human and camel strains from the 2012–2015 MERS outbreaks (Tai et al., 2016, 2017; Nyon et al., 2018). These RBD proteins maintain good conformation, functionality, antigenicity, and immunogenicity, with ability to bind the DPP4 receptor and RBD-specific neutralizing mAbs and to elicit robust neutralizing antibodies cross-neutralizing multiple strains of MERS pseudoviruses and live MERS-CoV (Tai et al., 2016, 2017; Nyon et al., 2018). It is noted that the wild-type MERS-CoV RBD proteins consisting of the identified critical neutralizing domain confer partial protection of hDPP4-transgenic (hDPP4-Tg) mice from MERS-CoV infection without causing immunological toxicity or eosinophilic immune enhancement (Tai et al., 2016; Wang Y. et al., 2017; Nyon et al., 2018); nevertheless, a structurally designed mutant version of such RBD protein with a non-neutralizing epitope masked (T579N) preserves intact conformation and significantly improves overall neutralizing activity and protective efficacy, resulting in the full protection of hDPP4-Tg mice against high-dose MERS-CoV challenge (Du et al., 2016a).

The above studies indicate that protein lengths to be chosen as MERS-CoV subunit vaccines and/or structure-based vaccine design can impact on the immunogenicity and/or protection of RBD-based subunit vaccines.

#### MERS-CoV Subunit Vaccines Based on Non-RBD S Protein Fragments

MERS vaccines targeting non-RBD regions of S protein have been developed and investigated in mice and NHPs. It has been shown that a MERS-CoV S1 protein formulated with Ribi (for mice) or aluminum phosphate (for NHPs) adjuvant elicited robust neutralizing antibodies in mice and NHPs against divergent strains of pseudotyped and live MERS-CoV, protecting NHPs from MERS-CoV infection (Wang et al., 2015). In addition, MERS-CoV S1 protein adjuvanted with Advax and Sigma Adjuvant System induced low-titer neutralizing antibodies in dromedary camels with reduced and delayed viral shedding after MERS-CoV challenge, but high-titer neutralizing antibodies in alpacas with complete protection of viral shedding from viral infection, indicating that protection of MERS-CoV infection is positively correlated with serum neutralizing antibody titers (Adney et al., 2019). Moreover, immunization with a recombinant MERS-CoV NTD protein (rNTD) can induce neutralizing antibodies and cell-mediated responses, protecting Ad-hDPP4-transduced mice against MERS-CoV challenge (Jiaming et al., 2017). Notably, specific antibodies with neutralizing activity have been elicited by a S2 peptide sequence (residues 736–761) of MERS-CoV in rabbits (Yang et al., 2014a), but the protective efficacy of this peptide vaccine is unknown. The above reports demonstrate the potential for the development of MERS subunit vaccines based on the non-RBD fragments of MERS-CoV S protein.

#### MERS-CoV Subunit Vaccines Based on Non-S Structural Proteins

Unlike SARS subunit vaccines which have been designed based on viral N and M proteins, it appears that very few subunit vaccines have been developed based on the non-S structural protein(s) of MERS-CoV. One study reports the induction of specific antibodies by MERS-CoV N peptides (Yang et al., 2014a), and another report shows that N protein is used for development of vaccines based on viral vector Vaccinia virus, modified Vaccinia Ankara (MVA) (Veit et al., 2018). This may be potentially a consequence of the weak immunogenicity and/or protective efficacy of non-S structural proteins, further confirming the role of MERS-CoV S protein as the key target for the development of MERS vaccines, including subunit vaccines.

#### Potential Factors Affecting MERS-CoV Subunit Vaccines

Similar to SARS-CoV subunit vaccines, the immunogenicity and/or protection of MERS-CoV subunit vaccines may also be affected by a number of factors, such as antigen sequences, fragment lengths, adjuvants, vaccination pathways, antigen doses, immunization doses and intervals used.

As described above, MERS-CoV subunit vaccines containing different antigens or fragment lengths, particularly those based on the RBD, have apparently variable immunogenicity and/or protective efficacy, and a critical neutralizing domain that contains an RBD fragment corresponding to residues 377–588 of S protein elicits the highest neutralizing antibodies among several fragments tested (Ma et al., 2014b; Zhang et al., 2015).

Adjuvants play an essential role in enhancing host immune responses to MERS-CoV subunit vaccines, including those based on the RBD, and different adjuvants can promote host immune responses to variant levels (Lan et al., 2014; Zhang et al., 2016). For example, while a MERS-CoV RBD subunit vaccine (S377-588 protein fused with Fc) alone induced detectable neutralizing antibody and T-cell responses in immunized mice, inclusion of an adjuvant enhanced its immunogenicity. Particularly, among the adjuvants (Freund’s, aluminum, Monophosphoryl lipid A, Montanide ISA51 and MF59) conjugated with this RBD protein, MF59 could best potentiate the protein to induce the highest-titer anti-S antibodies and neutralizing antibodies, protecting mice against MERS-CoV infection (Zhang et al., 2016). Moreover, a recombinant RBD (rRBD) protein plus alum and CpG adjuvants elicited the highest neutralizing antibodies against pseudotyped MERS-CoV infection, whereas the strongest T-cell responses were induced by this protein plus Freund’s and CpG adjuvants (Lan et al., 2014).

Vaccination pathways are important in inducing efficient immune responses, and different immunization routes may elicit different immune responses to the same protein antigens. For example, immunization of mice with a MERS-CoV subunit vaccine (RBD-Fc) via the intranasal route induced higher levels of cellular immune responses and stronger local mucosal neutralizing antibody responses against MERS-CoV infection than those induced by the same vaccine via the S.C. pathway (Ma et al., 2014a). In addition, while Freund’s and CpG-adjuvanted rRBD protein elicited higher-level systematic and local IFN-γ-producing T cells via the S.C. route, this protein adjuvanted with Alum and CpG induced higher-level tumor necrosis factor-alpha (TNF-α) and interleukin 4 (IL-4)-secreting T cells via the I.M. route (Lan et al., 2014).

Antigen dosage, immunization doses, and intervals may significantly affect the immunogenicity of MERS-CoV subunit vaccines. Notably, a MERS-CoV RBD (S377-588-Fc) subunit vaccine immunized at 1 μg elicited strong humoral and cellular immune responses and neutralizing antibodies in mice although the one immunized at 5 and 20 μg elicited a higher level of S1-specific antibodies (Tang et al., 2015). In addition, among the regimens at one dose and two doses at 1-, 2-, and 3-week intervals, 2 doses of this protein boosted at 4 weeks resulted in the highest antibodies and neutralizing antibodies against MERS-CoV infection (Wang Y. et al., 2017).

## Potential Challenges and Future Perspectives for Sars-CoV and Mers-CoV Subunit Vaccines

Compared with other vaccine types such as inactivated virus and viral-vectored vaccines, SARS and MERS subunit vaccines are much safer and do not cause obvious side effects. However, these subunit vaccines may face some important challenges, mostly arising from their relatively low immunogenicity, which must be combined with appropriate adjuvants or optimized for suitable protein sequences, fragment lengths, and immunization schedules. In addition, structure and epitope-based vaccine design has become a promising strategy to improve the efficacy of subunit vaccines. This is evidenced by a structurally designed MERS-CoV RBD-based protein which has significantly improved neutralizing activity and protection against MERS-CoV infection (Du et al., 2016a). It is prospected that more structure-guided novel strategies will be developed to improve the overall immunogenicity and efficacy of subunit vaccines against emerging pathogenic human coronaviruses, including those targeting SARS-CoV and MERS-CoV. Although a large number of SARS and MERS subunit vaccines have been developed with potent immunogenicity and/or protection in available animal models, virtually all remain in the preclinical stage. It is thus expected that one or several of these promising subunit vaccines can be further processed into clinical trials to confirm their immunogenicity against viral infections in humans.

## Rapid Development of Subunit Vaccines Against the New Pathogenic Human Coronavirus

Currently, the newly identified 2019-nCoV is spreading to infect people, resulting in significant global concerns. It is critical to rapidly design and develop effective vaccines to prevent infection of this new coronavirus. Since S protein and its fragments, such as RBD, of SARS-CoV, and MERS-CoV are prime targets for developing subunit vaccines against these two highly pathogenic human CoVs, it is expected that similar regions of 2019-nCoV can also be used as key targets for developing vaccines against this new coronavirus (Jiang et al., 2020). Similarly, other regions of 2019-nCoV, including S1 and S2 subunits of S protein and N protein, can be applied as alternative targets for vaccine development. Taken together, the approaches and strategies in the development of subunit vaccines against SARS and MERS described in this review will provide important information for the rapid design and development of safe and effective subunit vaccines against 2019-nCoV infection.

## Author Contributions

SJ and LD conceived the idea and revised and edited the manuscript. NW and LD collected information and drafted the manuscript. JS performed the structural analysis. All authors read and made final approval of the manuscript.

## Conflict of Interest

The authors declare that the research was conducted in the absence of any commercial or financial relationships that could be construed as a potential conflict of interest.

## Footnotes

1 https://www.who.int/emergencies/mers-cov/en/

2 https://www.who.int/docs/default-source/coronaviruse/situation-reports/20200219-sitrep-30-covid-19.pdf?sfvrsn=6e50645_2

3 https://www.who.int/docs/default-source/coronaviruse/situation-reports/20200204-sitrep-15-ncov.pdf?sfvrsn=88fe8ad6_2

4 https://clinicaltrials.gov/ct2/show/NCT03615911

5 https://clinicaltrials.gov/ct2/show/NCT03399578

## Supplementary Material

FIGURE S1 Structures of SARS-CoV and MERS-CoV S proteins, RBDs, and their complexes with respective receptor. Trimeric S proteins of SARS-CoV (PDB: 5×5b) **(A)** and MERS-CoV (PDB: 5×5f) **(C)** are colored differently for each monomer. Two conformations of the RBD in each trimeric S protein are labeled as standing and lying states. The RBDs of each S protein are shown as light blue on the right panel. ACE2 and DPP4 receptors are respectively modeled to the trimeric S proteins by match-alignment of SARS-CoV RBD-ACE2 complex (PDB: 2AJF) to SARS-CoV S trimer **(B)** or MERS-CoV RBD-DPP4 complex (PDB: 4kr0) to MERS-CoV S trimer **(D)**. Each of the RBD-receptor complexes is shown on the right panel. ACE2, angiotensin-converting enzyme 2; DPP4, dipeptidyl peptidase 4; RBD, receptor-binding domain; S, spike.
