# Supplementary figures and images for: Subunit Vaccines Against Emerging Pathogenic Human Coronaviruses
Source: Front Microbiol. 2020 Feb 28;11:298. doi: 10.3389/fmicb.2020.00298 (PMC7105881; doi:10.3389/fmicb.2020.00298)

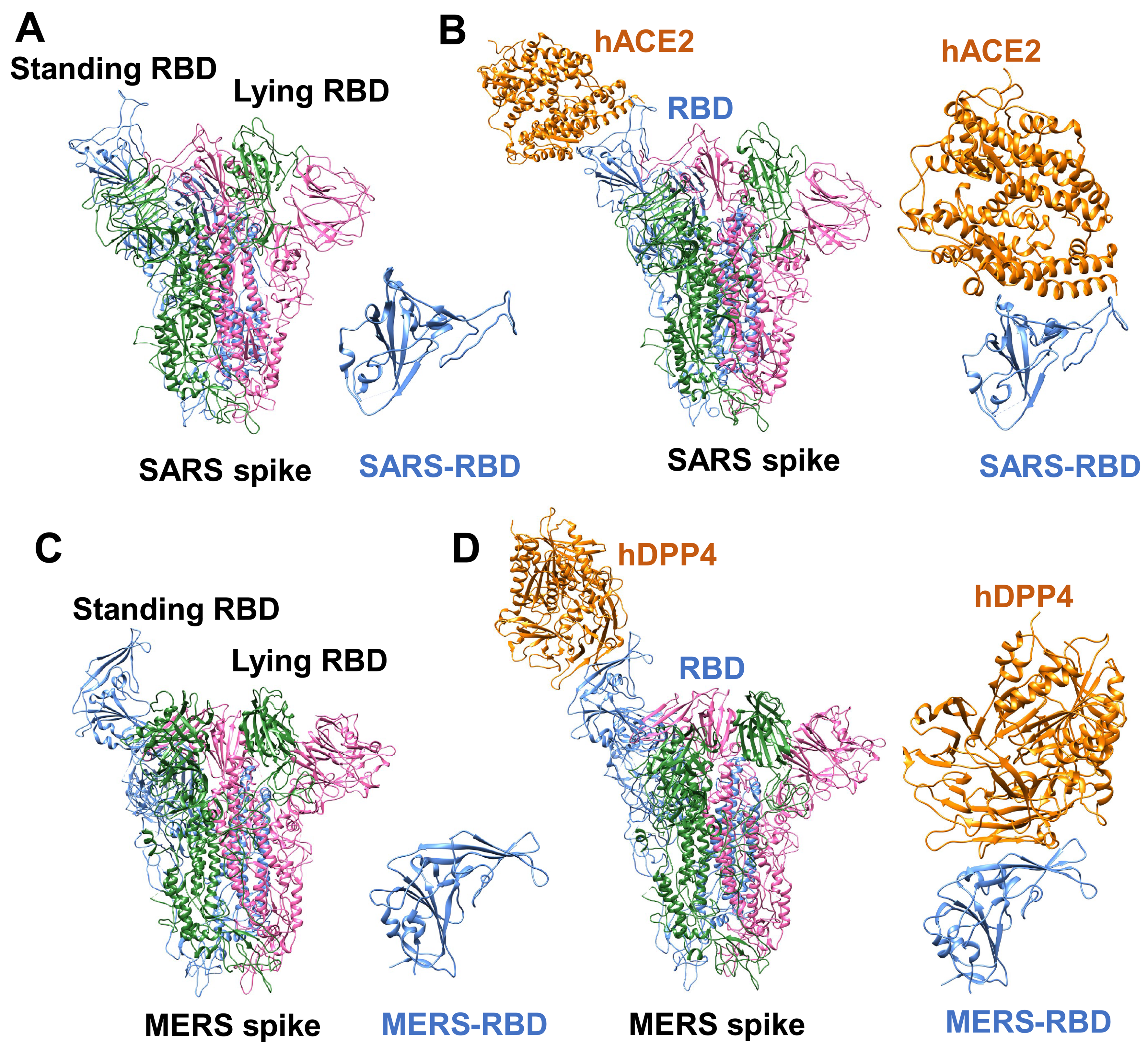

Supplement: FIGURE S1 — Structures of SARS-CoV and MERS-CoV S proteins, RBDs, and their complexes with respective receptor. Trimeric S proteins of SARS-CoV (PDB: 5×5b) (A) and MERS-CoV (PDB: 5×5f) (C) are colored differently for each monomer. Two conformations of the RBD in each trimeric S protein are labeled as standing and lying states. The RBDs of each S protein are shown as light blue on the right panel. ACE2 and DPP4 receptors are respectively modeled to the trimeric S proteins by match-alignment of SARS-CoV RBD-ACE2 complex (PDB: 2AJF) to SARS-CoV S trimer (B) or MERS-CoV RBD-DPP4 complex (PDB: 4kr0) to MERS-CoV S trimer (D). Each of the RBD-receptor complexes is shown on the right panel. ACE2, angiotensin-converting enzyme 2; DPP4, dipeptidyl peptidase 4; RBD, receptor-binding domain; S, spike. [file Image_1.TIF]
